# Chorus Size Shapes Calling Effort in the Asiatic Toad (*Bufo gargarizans*)

**DOI:** 10.3390/ani16203187

**Published:** 2026-10-11

**Authors:** Hua-Li Hu, Zhi-Qiang Chen, Jing Li, Wei-Cheng Zheng, Guo-Hua Ding

**Affiliations:** 1 College of Agriculture and Biotechnology, Lishui University, Lishui 323000, China; hualihu@njfu.edu.cn; 2 College of Life Sciences, Nanjing Forestry University, Nanjing 210037, China; 3 Administration Center of Zhejiang Jiulongshan National Nature Reserve, Suichang, Lishui 323000, China; 4 Yueqing Animal Disease Prevention and Control Center, Yueqing 325600, China; zqchen960717@foxmail.com; 5 Zhejiang Environmental Monitoring Engineering Co., Ltd., Hangzhou 311100, China; lijing0918@foxmail.com; 6 Suichang County Ecological Forestry Development Center, Suichang, Lishui 323000, China

**Keywords:** acoustic communication, behavioral plasticity, *Bufo gargarizans*, call composition, chorus size, explosive breeding

## Abstract

Acoustic communication is a core component of reproductive behavior in anuran amphibians. Among the factors shaping male calling behavior, the social acoustic environment plays a decisive role, primarily through chorus size; yet its regulatory effect in explosive-breeding species remains poorly understood. To investigate how chorus size affects the calling behavior of the Asiatic toad (*Bufo gargarizans*), we conducted a controlled experiment with two chorus-size treatments, small (five individuals per box) and large (15 individuals per box), corresponding to densities of approximately 13 and 39 individuals/m^2^, each replicated across three nights using unique individuals. We compared calling activity, call composition, and acoustic parameters between treatments. Total call number was significantly higher in large choruses, and the increase was not directly proportional to the number of males present, rising by a factor of 4.2 when group size was tripled. The proportion of two-note calls also increased, with a large effect size, although this difference was not statistically significant after multiple-comparison correction at the present sample size. None of the four acoustic parameters (call duration, dominant frequency, note duration, and note interval) showed a detectable response to chorus size, a result confirmed by mixed models fitted to all 401 recorded calls. Dominant frequency was the most conservative parameter, consistent with morphological constraint and stabilizing selection by female choice. We also show that the coefficient of variation of call duration is inflated when calls differing in note number are pooled, indicating that this metric should instead be assessed within note-number classes. Our study provides the first quantitative description of the advertisement call of *B. gargarizans* and indicates that males respond to a crowded acoustic environment primarily by increasing calling effort rather than by adjusting the acoustic structure of individual calls.

## Simple Summary

Frogs and toads generally gather in large groups during the breeding season as males call to attract mates. As more males gather, the acoustic environment becomes noisier and competition among them intensifies. Using the Asiatic toad as a model, we evaluated the effect of chorus size on male calling behavior. Males in large choruses called substantially more often, with the increase exceeding what would be expected from having males density alone. They also tended to produce shorter, two-note calls. In contrast, the basic acoustic features of individual calls, such as frequency and duration, showed no detectable change regardless of chorus size. This suggests that Asiatic toads cope with crowding and competition mainly by adjusting how often and in what form they call, rather than by changing the sound itself. These findings help us understand how animals flexibly adjust their behavior to cope with changes in their social environment.

## 1. Introduction

Acoustic communication is among the most essential and widespread modes of information transfer in anuran amphibians [1,2]. By producing species-specific calls, males not only convey species identity for interspecific recognition [3] but also broadcast information about body size and fighting ability [4,5,6], mating experience [7], and spatial location [8]. Following the classification framework of Toledo et al. [9], Köhler et al. [2] grouped anuran calls can be divided into four categories: feeding, aggressive, reproductive, and defensive calls. Among these, the advertisement call, a type of reproductive call, is one of the most frequently produced and best-studied call types in anurans [10,11,12], serving two central functions: attracting mates and signaling territory ownership to conspecifics [10,13]. Advertisement calls therefore play a pivotal role in reproductive success and population persistence, making the characterization of their acoustic features and regulatory mechanisms ecologically important for understanding anuran reproductive strategies.

Advertisement call characteristics are jointly shaped by intrinsic and extrinsic factors. Intrinsically, body size [5,14] and hormonal state [15] are primary determinants of call parameters. Extrinsically, abiotic factors such as temperature, humidity, and geographic location, as well as background noise, can induce adaptive adjustments in calling [14,16,17,18]. Beyond these abiotic factors, the social environment represents a key biotic influence that, despite long-standing attention, remains poorly understood. Chorus size and the local density it creates are core aspects of the social environment and can markedly affect male calling activity and call characteristics [19,20]: as choruses grow larger, individuals experience intensified male–male competition and acoustic interference, which may drive chorus-size-dependent adjustments in calling behavior [21]. However, responses to this social pressure vary among species, and the general patterns and underlying mechanisms remain unclear.

Analyses of anuran advertisement calls typically address spectral and temporal acoustic parameters [12,14,22]. A call is structurally composed of notes: it may consist of a single or several notes separated by regular intervals [2]. The two classes of parameters differ systematically in their variability. Under Gerhardt’s [23] static/dynamic hypothesis, spectral parameters—such as dominant frequency—are constrained by the morphology of the vocal apparatus and are relatively stable (static), whereas temporal parameters—such as call duration and note number—are readily influenced by environmental and social factors and are more variable (dynamic). Variability is commonly quantified by the coefficient of variation (CV). The static or dynamic status of a given parameter is not fixed across species, however, and therefore needs to be established for each species under a defined social context.

These effects are particularly pronounced in explosive-breeding species. Most anurans adopt an explosive breeding strategy, in which males form large chorus aggregations within a brief temporal window [24,25]. This strategy can enhance signal transmission and attract more females through the “chorus effect” [26,27], but it also produces an acoustically crowded environment in which individuals face intensified male–male competition, stronger intraspecific acoustic interference, and higher predation risk [28,29]. The level at which individuals respond to this selective pressure remains inconsistent across studies: some report that increasing chorus size primarily elevates calling activity and alters call composition [21,30], whereas others show that individuals adjust acoustic parameters (such as dominant frequency, temporal structure, or call rate) to improve signal detectability [17,31,32]. This discrepancy suggests that responses at the behavioral (calling activity and call composition) and acoustic-parameter levels may be governed by different selective pressures, and controlled experiments are needed to disentangle them.

The Asiatic toad (*Bufo gargarizans*), one of the most widely distributed bufonids in China [33], exhibits a typical explosive breeding pattern [34], making it a suitable model for studying how social acoustic environment shapes calling behavior. To our knowledge, however, its advertisement call has not been quantitatively described, and no experimental study has examined how chorus size affects its call features. We therefore manipulated chorus-size to compare the behavioral characteristics and acoustic parameters of *B. gargarizans* advertisement calls between chorus-size treatments. Specifically, we addressed two key questions: (1) Does chorus size alter male calling activity and call composition? (2) Does chorus size alter the acoustic parameters of advertisement calls?

## 2. Materials and Methods

### 2.1. Animal Collection and Husbandry

**Figure 1:**
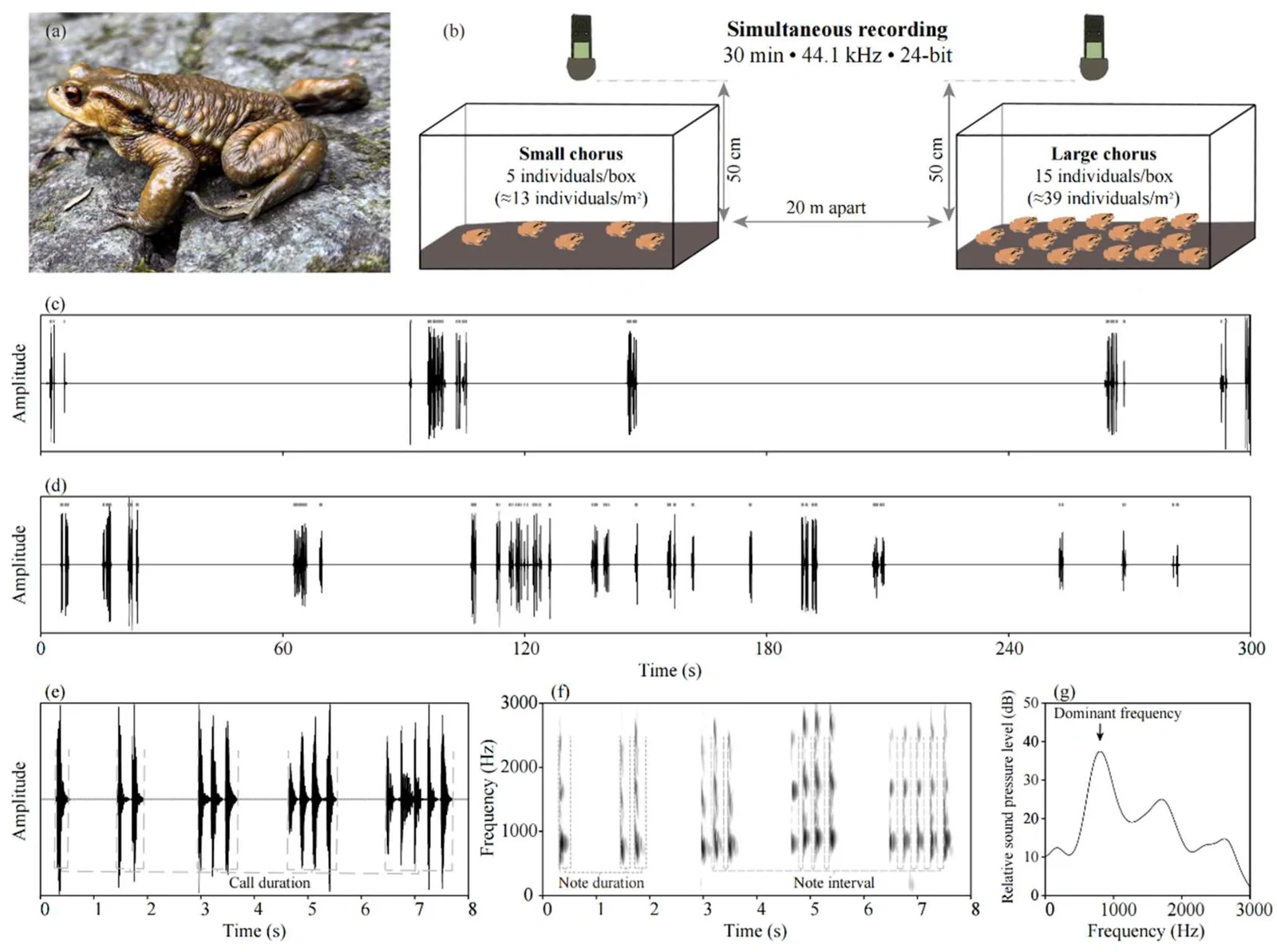
Acoustic structure of advertisement calls in *B. gargarizans*. (**a**) An adult male *B. gargarizans* from Huangshayao Town, Suichang County, Lishui, China (photo by Hua-Li Hu). (**b**) Experimental setup (drawn by Zi-Mu Ding): two identical boxes placed 20 m apart, holding 5 (small chorus) or 15 (large chorus) males, corresponding to approximately 13 and 39 individuals/m^2^, respectively. Because box area was fixed, male number and density were confounded and should not be conflated. A recorder was fixed 50 cm above the box floor, and both boxes were recorded simultaneously for 30 min. (**c**,**d**) Oscillograms showing the temporal distribution of calls within a representative 300 s segment of the 30 min recording in the (**c**) small-chorus and (**d**) large-chorus groups, with gray ticks above marking the position of each call. (**e**) Representative oscillograms of calls containing 1–5 notes, with gray dashed lines indicating call duration. (**f**) Corresponding spectrogram, with gray dashed lines indicating note duration and note interval. (**g**) Power spectrum, with dominant frequency marked by the arrow.

In February 2019, we collected 60 adult male *B. gargarizans* during the breeding season at Huangshayao Town, Suichang County, Zhejiang Province, China (28°24′35″ N, 118°48′36″ E) (body length 80.6 ± 6.4 mm, body mass 51.1 ± 12.0 g; mean ± SD) (Figure 1a). Field collection was permitted by the Suichang County Ecological Forestry Development Center. The toads were transported to the herpetological laboratory of Lishui University in Liandu District, Lishui City (approximately 60 km from the collection site) and housed in an outdoor enclosure (200 × 150 × 110 cm) lined with a 5 cm layer of moist coconut coir. A water basin and curved tiles provided unrestricted access to water and shelter, respectively. During the one-week acclimation period, toads were exposed to natural outdoor photoperiod and ambient temperature, with relative humidity maintained above 70%. Individuals were fed mealworms and crickets regularly. As this study involved only non-invasive acoustic recording of calling behavior, no invasive or painful procedures were performed. To minimize stress, the enclosure simulated the toads’ natural breeding microhabitat (moist coconut coir, shallow water, curved tile shelters, relative humidity > 70%). After the experiment, all individuals were released back to the collection site.

### 2.2. Experimental Design and Recording

To examine the effect of chorus size on the calling behavior of *B. gargarizans*, we established two chorus-size treatments comprising 5 (small) and 15 (large) males per chorus group. Each treatment group was placed in one of two identical plastic boxes (length × width × height = 74 × 52 × 50 cm) during the experiments. Given the box floor area of 0.38 m^2^, the two treatments corresponded to densities of approximately 13 and 39 individuals/m^2^, which span the range from sparse, dispersed calling to dense chorus aggregation typical of explosive breeding events. Because the box floor area was fixed, manipulating chorus size inevitably also changed density; male number and density are therefore confounded in this design and should not be conflated. The chorus sizes tested in our study align with those used in previous anuran studies [35,36] and reflect natural breeding aggregations of *B. gargarizans* while avoiding excessive crowding that could otherwise induce stress and alter male calling behavior [37]. Each box contained a 2 cm layer of moist coconut coir and a 1–2 cm layer of shallow water to simulate the natural breeding microhabitat of *B. gargarizans* and support normal reproductive calling. Each box was sprayed with water before each night’s trial to maintain relative humidity above 80%; no spraying was carried out during the recording sessions. To prevent spatial segregation and male–male competition over shelter occupancy from confounding calling behavior, tile shelters were not provided for the experimental boxes [38]. Experiments for the small- and large-chorus treatments were run simultaneously to standardize environmental conditions. Both boxes were placed 20 m apart to reduce acoustic interference between treatments. The spatial positions of the two chorus-size treatments were randomly assigned to prevent positional bias. According to records from the Liandu District meteorological station (Lishui, Zhejiang Province, China), daily air temperature on the three experimental nights ranged from 5 to 11 °C, with light winds (Beaufort force 1–2) and no precipitation occurring during the recording sessions. Background noise and call amplitude were not measured in the field but extracted post hoc in Praat from five randomly selected positions per recording: background noise was read from silent intervals between calls, and call amplitude from calling segments. These uncalibrated relative values were used only for between-treatment comparison. A Welch’s *t*-test showed no significant difference between treatments in background level (57.2 ± 2.1 dB) or signal-to-noise ratio (SNR, 22.2 ± 0.8 dB) (all *p* > 0.22); however, given the small sample size (*n* = 3 per treatment) and the fact that these variables were not experimentally manipulated, this test cannot be taken as evidence of no difference, and the analysis therefore necessarily assumes that there were no systematic differences between treatments in these variables.

The experiment was conducted over three consecutive nights. A schematic of the experimental setup is shown in Figure 1b. Experiments began at 19:00, where individuals were randomly assigned to either the small-chorus group (5 individuals) or large-chorus group (15 individuals). After 1 h of acclimation, both boxes were recorded simultaneously for 30 min (20:00–20:30), after which the individuals were returned to the housing enclosure. A total of 60 different individuals were used across the experiment, with each toad tested only once. Individuals tested on a given night were subsequently isolated in separate holding pools. The three nights therefore constituted three independent replicates, with no individuals sampled more than once. Acoustic signals were recorded using a digital recorder (ICD-SX734, Sony Corporation, Tokyo, Japan) fixed 50 cm above the box floor, with the microphone oriented toward the box. Recordings were saved as WAV files (PCM format; 44.1 kHz sampling rate; 24-bit resolution; built-in noise-reduction enabled). A total of three complete audio files per chorus-size treatment were obtained.

### 2.3. Acoustic Analysis and Statistics

The two-channel raw recordings were merged into a single channel in Praat 6.1.16. All complete calls in each recording were manually identified and labeled from oscillograms and spectrograms by two independent observers (Figure 1c,d). A call was defined as a continuous unit composed of notes separated by regular intervals; a prolonged silence marked the end of one call and the beginning of the next. For each recording, we counted the total call number and calculated group-level average calls per individual (total calls divided by the number of males in each trial), representing a box-level mean as calls could not be assigned to specific individuals. Calls were classified by note number (hereafter note-number classes, one to five notes), and the relative proportion of calls with each note number was computed for each recording.

Following Köhler et al. [2], we measured four acoustic parameters including call duration, dominant frequency, note duration, and note interval (Figure 1e–g). Dominant frequency was measured from the power spectrum in Praat using a 512-point fast Fourier transform (FFT). For each recording, the mean of each acoustic parameter was used as the unit of analysis, and all between-treatment comparisons were performed at the recording (box-night) level (*n* = 3 per treatment) rather than on individual calls. In addition, the coefficient of variation (CV = SD/mean × 100%) of each parameter was calculated to assess within-group variability in acoustic parameters.

All statistical analyses were conducted in R 4.5.1. Due to the limited number of replicates per chorus-size treatment (*n* = 3), between-treatment differences were compared using Welch’s *t*-tests. These tests were applied to total call number, group-level average calls per individual, the relative proportion of calls in each note-number class, and the four acoustic parameters and their CVs. Given the limited statistical power inherent to this sample size, we additionally report Hedges’ g with 95% confidence intervals (CIs) for all between-treatment comparisons and interpret non-significant results as an absence of detectable differences rather than as evidence of equivalence.

To test whether the acoustic parameters differed in their degree of variability, as predicted by the static/dynamic acoustic-property hypothesis, we compared the CV of the four acoustic parameters. As each recording yielded a CV value for each acoustic parameter, these values were not statistically independent; we therefore treated acoustic parameter as a within-subject factor and recording as the blocking unit. CV values were log-transformed to stabilize pronounced variance heterogeneity across parameters and analyzed using a repeated-measures ANOVA. Sphericity was assessed using Mauchly’s test, with sphericity defined as the assumption that the variances of the differences between all possible pairs of repeated measurements are equal. Although sphericity was not violated according to Mauchly’s test, the Greenhouse–Geisser-corrected results were also reported as a conservative check, followed by pairwise paired *t*-tests with Bonferroni adjustment. A Friedman test was also conducted as a non-parametric robustness check. Before combining data across chorus-size treatments, we verified that the CV of each acoustic parameter did not differ between treatments using Welch’s *t*-test. Parameters were additionally classified using Gerhardt’s [23] descriptive criteria (static, CV < 5%; intermediate, 5–12%; dynamic, CV > 12%).

Call duration was associated with note number together with the duration of, and the intervals between, its constituent notes. Its CV computed across all calls therefore conflates variation in note number with variation in the temporal structure of calls. To separate these two sources, we examined the correlation between measured call duration and its reconstructed value (note number × note duration + (note number − 1) × note interval) and recomputed the CV of call duration separately within each note-number class, using only recording × note-number combinations containing at least 5 calls.

To complement the recording-level analyses, we also fitted linear mixed models to all recorded calls (*n* = 401) using the R package ‘lme4’, with chorus size as a fixed effect and recording as a random intercept, which accounts for the nesting of calls within recordings. Separate models were fitted for call duration, dominant frequency, note duration and note interval. Because call duration depends strongly on the number of notes a call contains, note number was included as a covariate in that model. Note number itself was modeled using a Poisson generalized linear mixed model (GLMM). Because the treatments differed in the distribution of note numbers rather than in their mean, a cumulative link mixed model (CLMM) treating note number as an ordinal response was also fitted using the R package ‘ordinal’. Degrees of freedom for fixed effects were approximated by Satterthwaite’s method using the R package ‘lmerTest’.

Statistical significance was set at α = 0.05. A Bonferroni-corrected threshold (α = 0.010) was applied to the five comparisons of note-number classes, which address a single question and therefore constitute a family of tests. Total call number and the group-level average calls per individual were not corrected in this way, as the latter is derived from the former and the two do not represent independent hypotheses. The four acoustic parameters and their CVs were likewise evaluated at α = 0.05; all of these comparisons were non-significant before correction, so correction would not alter the conclusions. Data are presented as mean ± standard deviation (SD).

## 3. Results

We recorded a total of 401 valid advertisement calls in *B. gargarizans* (large-chorus group: 324 calls; small-chorus group: 77 calls). In both chorus-size treatments, *B. gargarizans* produced advertisement calls containing 1–5 notes (Figure 1e,f). Across all recordings, calls had a mean duration of 0.669 ± 0.145 s, a dominant frequency of 838.4 ± 27.6 Hz, a note duration of 0.124 ± 0.022 s and a note interval of 0.170 ± 0.038 s.

**Figure 2:**
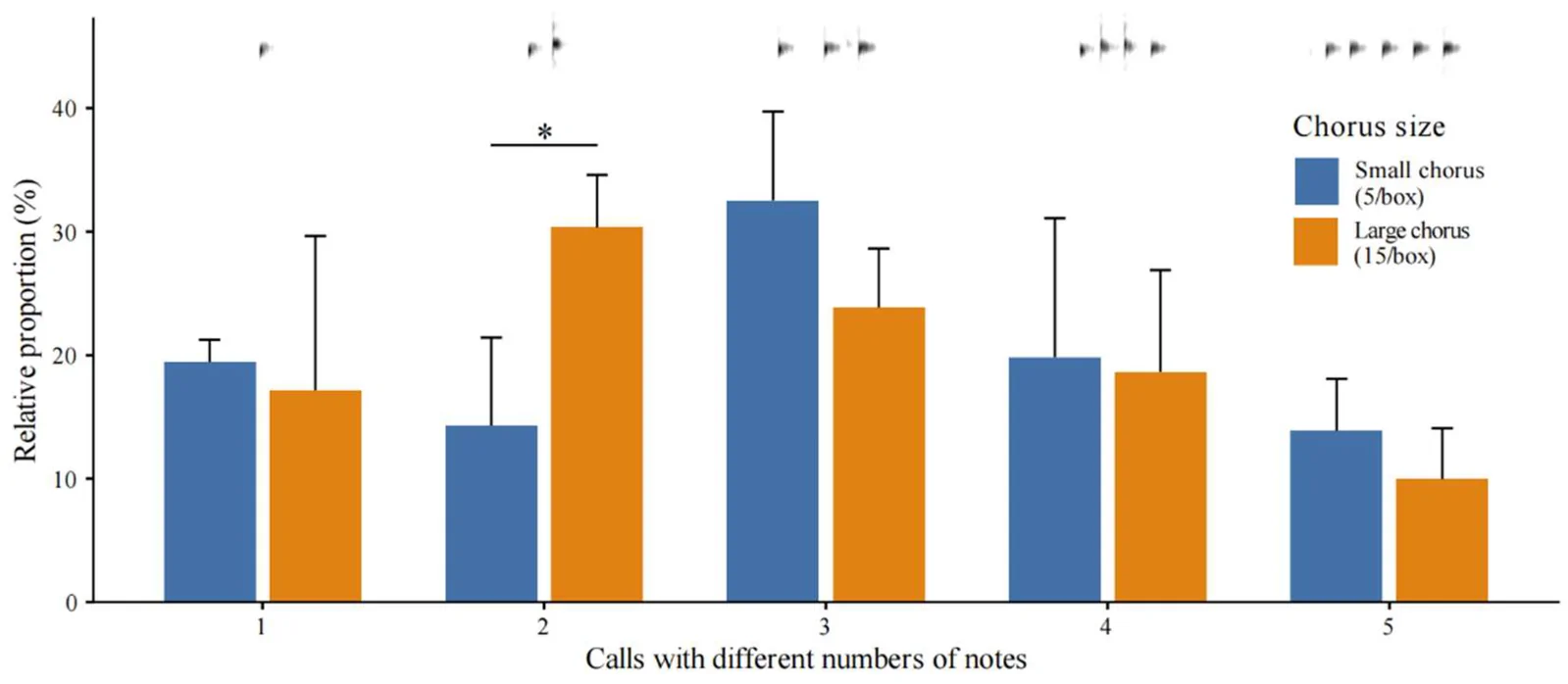
Relative proportions of calls with different numbers of notes in *B. gargarizans* under two chorus-size treatments. Spectrograms above each group show representative calls with the corresponding number of notes (1–5). Bars show mean + SD. The asterisk indicates a difference at uncorrected *p* < 0.05.

Call composition differed between the two chorus-size treatments. Two- and three-note calls predominated in both treatments. The relative proportion (%) of two-note calls was higher in the large-chorus group (30.3 ± 4.3) than in the small-chorus group (14.3 ± 7.1) (*t* = 3.35, df = 3.26, *p* = 0.039, Hedges’ g = 2.19, 95% CI [−0.11, 4.48]; Figure 2), whereas the proportions of one-, three-, four-, and five-note calls did not differ between treatments (all *p* ≥ 0.167; Figure 2). Because five note-number classes were compared, we applied a Bonferroni-corrected threshold (α = 0.010). Under this criterion, the difference in two-note calls was not statistically significant, despite a large effect size. The increase in two-note calls was accompanied by a non-significant decrease in three-note calls (23.9 ± 4.4% vs. 32.5 ± 6.9%, *p* = 0.167). Consistent with a distributional shift rather than a change in central tendency, the mean number of notes per call did not differ between treatments (Poisson GLMM, β = −0.078, *p* = 0.475; CLMM, β = −0.353, *p* = 0.394).

**Figure 3:**
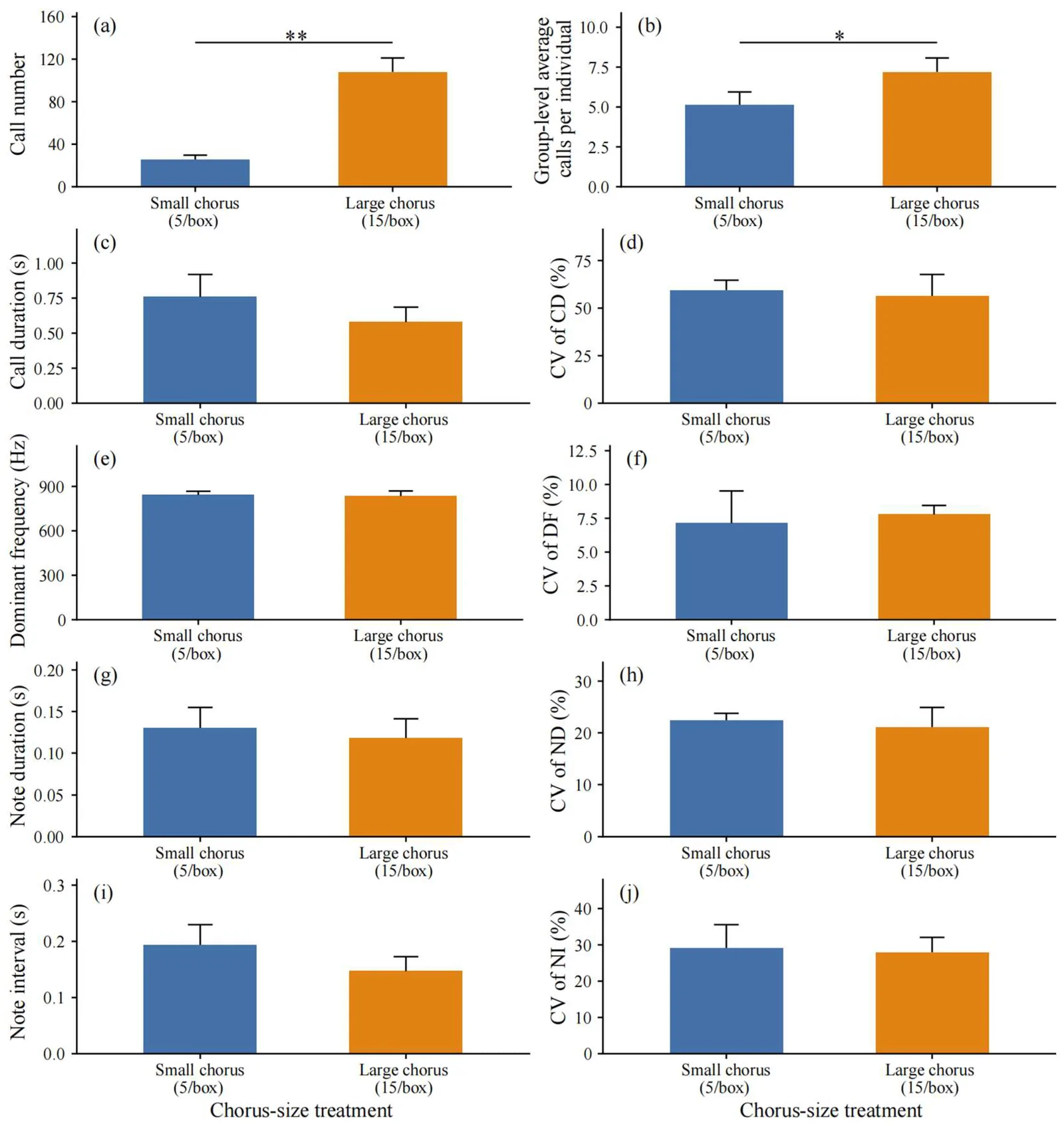
Call number, acoustic parameters, and their coefficients of variation (CV) in *B. gargarizans* under two chorus-size treatments. (**a**) Call number; (**b**) group-level average calls per individual; (**c**) call duration (CD); (**d**) CV of CD; (**e**) dominant frequency (DF); (**f**) CV of DF; (**g**) note duration (ND); (**h**) CV of ND; (**i**) note interval (NI); (**j**) CV of NI. Bars show mean + SD. Asterisks indicate significant differences between treatments (* *p* < 0.05; ** *p* < 0.01).

Calling activity was higher in the large-chorus group. The total call number was 108.0 ± 13.1 in the large-chorus group and 25.7 ± 4.0 in the small-chorus group (*t* = 10.42, df = 2.38, *p* = 0.005, g = 6.81 [2.08, 11.53]; Figure 3a), and the group-level average calls per individual was 7.2 ± 0.9 and 5.1 ± 0.8, respectively (*t* = 3.01, df = 3.98, *p* = 0.040, g = 1.97 [−0.24, 4.18]; Figure 3b).

No significant effect of chorus size was detected for any of the four acoustic parameters (all *p* ≥ 0.149; Figure 3c,e,g,i). Given the small number of independent replicates (*n* = 3 per treatment), these results indicate an absence of detectable differences rather than evidence of stability. Effect sizes varied among parameters: dominant frequency and note duration showed negligible to small differences (g = 0.21 [−1.61, 2.02] and g = 0.41 [−1.42, 2.24], respectively), whereas call duration and note interval showed large but non-significant differences, with shorter values in large choruses (g = 1.06 [−0.88, 2.99] and g = 1.20 [−0.77, 3.16], respectively). The shorter mean call duration in large choruses (0.579 ± 0.107 s vs. 0.758 ± 0.160 s) is consistent with the concurrent shift toward two-note calls, since call duration is constrained by note number. Linear mixed models fitted to all 401 calls with recording as a random intercept gave the same conclusion: chorus size had no detectable effect on note duration (β = −0.012, *p* = 0.569), note interval (β = −0.046, *p* = 0.142), dominant frequency (β = −8.06, *p* = 0.766), or call duration after controlling for note number (β = −0.123, *p* = 0.176).

The CVs of these parameters did not differ between treatments (all *p* ≥ 0.620, all |g| ≤ 0.37; Figure 3d,f,h,j), and the two treatments were therefore combined for the following analysis. The CVs differed among the four acoustic parameters (repeated-measures ANOVA on log-transformed CVs: *F*_3,15_ = 207.55, *p* < 0.001, generalized eta squared (ges) = 0.96; Mauchly’s test W = 0.40, *p* = 0.65; Greenhouse–Geisser-corrected *p* < 0.001; and Friedman test χ^2^ = 17.0, *p* < 0.001). Specifically, the CV was highest for call duration (57.9 ± 8.0%) and lowest for dominant frequency (7.5 ± 1.6%). The CVs of note interval (28.6 ± 4.9%) and note duration (21.8 ± 2.7%) were intermediate and did not significantly differ from each other (P_adj_ = 0.201). In contrast, all other pairwise comparisons were significant (all *p* < 0.01). Following Gerhardt’s descriptive criteria, dominant frequency fell within the intermediate range (5–12%) and approached the static end of the continuum, whereas note duration, note interval, and call duration were classified as dynamic (CV > 12%).

**Figure 4:**
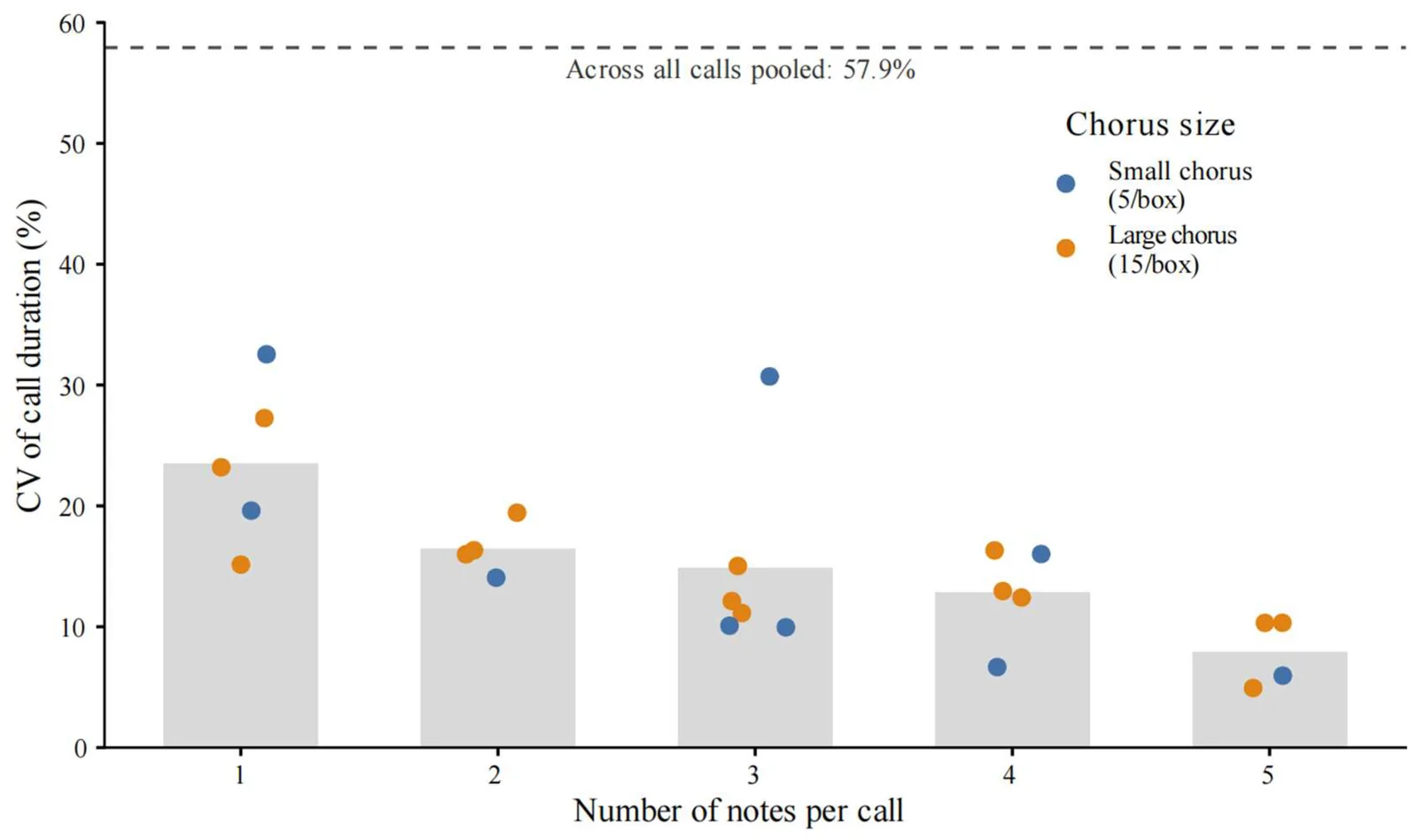
Coefficient of variation (CV) of call duration in *B. gargarizans*, computed within fixed note-number classes. Gray bars show the mean CV across recordings for each note-number class. Individual points represent individual recordings, colored by chorus-size treatment. Only recording × note-number combinations containing at least five calls are included. The dashed line indicates the CV of call duration computed across all calls irrespective of note number.

The high CV of call duration was largely attributed to variation in note number rather than plasticity in temporal structure. Measured call duration was almost perfectly predicted by its reconstructed value from note number, note duration, and note interval (r = 0.999), indicating that call duration is a composite rather than an independent acoustic parameter. Recomputed within fixed note-number classes, the CV of call duration fell from 57.9% to 23.6% (one-note calls), 16.5% (two-note), 14.8% (three-note), 12.9% (four-note), and 7.9% (five-note calls), values within the range observed for note duration and note interval (Figure 4). The high CV computed across all calls therefore reflects the inclusion of calls differing in note number within a single estimate. Among the parameters structurally independent of note number, dominant frequency was the least variable, while note duration and note interval showed comparable and substantially higher variability.

## 4. Discussion

In this study, the total call number was significantly higher in the large-chorus group than in the small-chorus group, confirming that chorus size modulates calling behavior in *B. gargarizans*. Similarly, group-level average calls per individual showed the same pattern, with a large effect size, though the two measures are not independent. Notably, although more males were present in the large-chorus group, the increase in total call output was not directly proportional to the increase in male number: tripling group size raised total call number by a factor of 4.2, indicating that the effect cannot be attributed to the presence of more males alone.

This pattern is consistent with previous studies in which male calling activity increases with chorus size or the presence of competitors [18,19], although the direction of the response varies among species. Such socially facilitated calling behavior indicates that the acoustic environment of a chorus can strongly modulate reproductive activity and is closely tied to the breeding tactics of explosive-breeding anurans. In these species, males in dense aggregations typically adopt a scramble-competition mating strategy, and increasing chorus size intensifies male–male competition, prompting males to raise their calling rate to enhance signal competitiveness [23,39,40]. Physiologically, this competition-driven escalation may be mediated by hormonal regulation: the social acoustic stimulation of a chorus can activate the hypothalamic–pituitary–gonadal axis and promote testosterone secretion, thereby increasing calling effort [41,42,43,44]. For example, circulating testosterone is positively associated with calling performance in *Hyla chrysoscelis* [44], but this has not been demonstrated in *B. gargarizans*.

The proportion of two-note calls was higher in the large-chorus treatment, with a large effect size, although this difference was not statistically significant after correction for multiple comparisons across the five note-number classes. The mechanism underlying this shift is not fully understood, but two non-mutually exclusive hypotheses may offer partial explanations, both of which warrant further testing. The first is an energy trade-off hypothesis: two-note calls contain fewer notes and are consequently shorter, thus incurring a lower energetic cost per call than multi-note calls. Advertisement calling is among the most energetically demanding aerobic activities in anurans, with metabolic rates elevated several to tens of times above resting level [45]. As calling effort in a large chorus draws on limited energetic reserves, and endurance rivalry over sustained chorus attendance strongly predicts male mating success [46], shifting toward shorter two-note calls may maintain acoustic presence while lowering the cost per call. We note, however, that the mean number of notes per call did not decrease, and the proportions of four- and five-note calls were unchanged; the shift was largely a redistribution between two- and three-note calls. Consequently, any energetic savings would likely be modest, and confirming this hypothesis will require direct measurement of calling metabolic rate. The second is an acoustic time-competition hypothesis: in crowded acoustic environments, signal overlap intensifies, and shorter calls are more easily transmitted within gaps between competitors’ calls, thus reducing the likelihood of masking [36,47]. Consistent with this hypothesis, increasing chorus size in túngara frogs elevated the risk of acoustic overlap, leading males to overwhelmingly produce short two-chuck calls even in the largest choruses [18]. Similarly, in a treefrog, ambient noise impairing particular notes altered female preference and male–male responses [48]. Notably, in *Kurixalus odontotarsus* [49] and *Engystomops pustulosus* [50], escalating competition instead promotes calls with more notes, contrary to our finding, indicating that species differ substantially in how they adjust call composition under competition, that the mechanisms underlying these differences remain to be determined.

No detectable effect of chorus size was found for any of the four acoustic parameters, consistent with reports for other anurans in which chorus size did not influence call parameters [17]. Given the small number of replicates, however, this should be read as an absence of detectable differences rather than as demonstrated stability. The parameters nonetheless differed in how close they came to a null effect: dominant frequency and note duration showed negligible differences between treatments, whereas call duration and note interval showed large effect sizes reflecting shorter values in large choruses. These conclusions were supported by mixed models fitted to all 401 calls, which detected no effect of chorus size on any parameter. The conservatism of dominant frequency likely reflects its dual constraint by morphology and female choice. Because dominant frequency is negatively correlated with male body size, females can use it to assess mate size [5,6], which imposes stabilizing selection on this parameter [30] and makes it unlikely to be adjusted in response to short-term changes in the social environment.

The CV of dominant frequency (7.5%) was substantially lower than those of note duration (21.8%) and note interval (28.6%), conforming to Gerhardt’s static–dynamic framework [2,23] and consistent with findings reported in other anuran species [14,51,52,53,54]. Call duration showed the highest apparent variability; however, as noted above, this largely reflected variation in note number rather than plasticity in temporal structure. Among parameters independent of note number, dominant frequency was the most conservative. Together, these results indicate that *B. gargarizans* responds to increased chorus size primarily at the behavioral level rather than through fine adjustment of acoustic parameters.

Our analysis also carries a methodological implication. Because call duration is related to note number together with note duration and inter-note interval, computing its CV across calls of differing note number conflates two sources of variation. In *B. gargarizans*, this inflated the variability of call duration two- to seven-fold relative to values computed within fixed note numbers. In species whose calls vary in note number, the classification of call duration as a highly dynamic property may therefore partly be an artifact of pooling. Thus, we suggest that variability in call duration be assessed within note-number classes.

Our study has several limitations. First, group size and density were inherently confounded due to the fixed enclosure area. Consequently, the two treatments differed in male abundance and spatial density, and we therefore cannot separate their independent effects. This coupling mirrors natural explosive breeding aggregations, where larger choruses occur at higher local density, but a crossed design using enclosures of different sizes would be needed to disentangle group density from size. Second, the number of independent replicates was small (three nights per treatment), thus limiting statistical power. While the difference in total call output was large and robust, the non-significant results for acoustic parameters should be interpreted with caution. Third, several variables were not recorded at the time of the experiment: air temperature was not measured instrumentally at the enclosures, and individual identities were not tracked across specific box assignments on a given night. Nonetheless, both treatments were recorded simultaneously 20 m apart, with individuals randomly assigned and tested only once, so ambient conditions and body size were not systematically associated with either treatment. Furthermore, meteorological records indicate comparable conditions across the three nights of testing, and background noise and SNR, which were quantified post hoc from the recordings, could not be tested for treatment differences; the analysis therefore necessarily assumes that there were no systematic differences between treatments in these variables.

Our outdoor enclosures may also not fully capture chorus-size effects under natural breeding conditions, where chorus size covaries with abiotic factors such as temperature and background noise. Future work combining measurements of metabolic rate and hormone levels, and a multi-species comparative framework, would help test the proposed hypotheses and clarify the general and species-specific mechanisms of chorus-size-dependent calling in explosive-breeding anurans.

## 5. Conclusions

Using a chorus-size manipulation experiment, we provide the first quantitative description of the advertisement call of *B. gargarizans* and the first experimental evidence that chorus size shapes its calling behavior. Males in large choruses produced substantially more calls in total—an increase disproportionate to the number of males present—and the proportion of short two-note calls also rose, although the latter effect was not robust to correction for multiple comparisons at this sample size. None of the four acoustic parameters showed a detectable response to chorus size; dominant frequency was highly conservative, whereas call duration and note interval showed large but non-significant effects toward shorter values in large choruses. Taken together, our results indicate that *B. gargarizans* responds to an increasingly crowded acoustic environment primarily by increasing calling effort and adjusting call composition, while the spectral structure of the call remains constrained. Our findings provide experimental evidence demonstrating how the social acoustic environment regulates calling in an explosive-breeding anuran, establishing a foundation for further work on behavioral plasticity in dense breeding aggregations.

## Data Availability

All data supporting the findings of this study are available within the article and its Supplementary Materials (Tables S1 and S2).

## Supplementary Materials

The following supporting information can be downloaded at: https://www.mdpi.com/article/10.3390/ani16203187/s1, Table S1: Recording-level summary of advertisement call data in *Bufo gargarizans* under two chorus-size treatments; Table S2: Call-level raw data for advertisement call analysis in *Bufo gargarizans* under two chorus-size treatments.

## Abbreviations

The following abbreviations are used in this manuscript:

CD	call duration
DF	dominant frequency
ND	note duration
NI	note interval
CV	coefficient of variation
SD	standard deviation
FFT	fast Fourier transform
ANOVA	analysis of variance
HSD	honestly significant difference
GLMM	generalized linear mixed model
CLMM	cumulative link mixed model
SNR	signal-to-noise ratio
PCM	pulse-code modulation
CI	confidence interval
ges	generalized eta squared
